# NCBP2 and TFRC are novel prognostic biomarkers in oral squamous cell carcinoma

**DOI:** 10.1038/s41417-022-00578-8

**Published:** 2023-01-12

**Authors:** Rahul Arora, Logan Haynes, Mehul Kumar, Reid McNeil, Jahanshah Ashkani, Steven C. Nakoneshny, T. Wayne Matthews, Shamir Chandarana, Robert D. Hart, Steven J. M. Jones, Joseph C. Dort, Doha Itani, Ayan Chanda, Pinaki Bose

**Affiliations:** 1 Department of Biochemistry and Molecular Biology, Cumming School of Medicine, University of Calgary, Calgary, Canada; 2 Canada’s Michael Smith Genome Sciences Centre, Vancouver, BC Canada; 3 Ohlson Research Initiative, Arnie Charbonneau Cancer Institute, Cumming School of Medicine, University of Calgary, Calgary, Canada; 4 Department of Surgery, Section of Otolaryngology-Head & Neck Surgery, Cumming School of Medicine, University of Calgary, Calgary, Canada; 5 Department of Oncology, Cumming School of Medicine, University of Calgary, Calgary, T2N 4N1 AB Canada; 6 Department of Anatomic and Molecular Pathology, Dalhousie University, Saint John, NB Canada

**Keywords:** Biomarkers, Oral cancer, Oncogenes

## Abstract

There are few prognostic biomarkers and targeted therapeutics currently in use for the clinical management of oral squamous cell carcinoma (OSCC) and patient outcomes remain poor in this disease. A majority of mutations in OSCC are loss-of-function events in tumour suppressor genes that are refractory to conventional modes of targeting. Interestingly, the chromosomal segment 3q22-3q29 is amplified in many epithelial cancers, including OSCC. We hypothesized that some of the 468 genes located on 3q22-3q29 might be drivers of oral carcinogenesis and could be exploited as potential prognostic biomarkers and therapeutic targets. Our integrative analysis of copy number variation (CNV), gene expression and clinical data from The Cancer Genome Atlas (TCGA), identified two candidate genes: *NCBP2, TFRC*, whose expression positively correlates with worse overall survival (OS) in HPV-negative OSCC patients. Expression of *NCBP2* and *TFRC* is significantly higher in tumour cells compared to most normal human tissues. High NCBP2 and TFRC protein abundance is associated with worse overall, disease-specific survival, and progression-free interval in an in-house cohort of HPV-negative OSCC patients. Finally, due to a lack of evidence for the role of NCBP2 in carcinogenesis, we tested if modulating NCBP2 levels in human OSCC cell lines affected their carcinogenic behaviour. We found that NCBP2 depletion reduced OSCC cell proliferation, migration, and invasion. Differential expression analysis revealed the upregulation of several tumour-promoting genes in patients with high *NCBP2* expression. We thus propose both NCBP2 and TFRC as novel prognostic and potentially therapeutic biomarkers for HPV-negative OSCC.

## Introduction

Head and neck cancers are the sixth most common cancer worldwide, resulting in more than 700,000 newly diagnosed cases in 2020 and over 400,000 deaths [1]. The vast majority of head and neck cancers are squamous cell carcinomas and oral squamous cell carcinoma (OSCC) is the most common head and neck cancer [1]. Smoking and/or alcohol consumption are canonical risk factors for OSCC, increasing the chance of incidence by up to 30 times. However, the human papillomavirus (HPV) has recently emerged as a major causal agent [2, 3]. These two aetiologic subsets represent distinct pathologies. However, HPV-positivity is mostly limited to oropharyngeal cancers in the head and neck region and is uncommon in other anatomical subtypes, including OSCC [4].

OSCC patients exhibit significant symptom burden; primary treatment involves surgical resection, which is associated with high morbidity [5, 6]. Most OSCC patients are also treated with post-operative radiotherapy (PORT) and/or chemotherapy. These treatment modalities have an adverse impact on quality-of-life. There have been limited advances in OSCC management, with overall survival (OS) improving by only 5% in the last 20 years [5, 6]. Furthermore, OSCC prognostication and treatment selection still relies heavily on the tumour-node-metastasis (TNM) staging system, where tumour biology has limited impact on treatment decisions. Hence, identifying improved targeted treatments and prognostic markers are major priorities in OSCC.

Identification of novel oncogenic drivers may permit more precise treatment selection and reduce treatment-related morbidity in low-risk patients while improving the management of high-risk patients. Prior studies aiming to characterize the genomic landscape in HNSCC have reported that loss of function mutations in several tumour suppressor genes—such as *TP53*, *NOTCH1*, and *CDKN2A*—drive carcinogenesis in a majority of cases [7]. However, tumour suppressor alterations are challenging drug targets because it is difficult to restore gene function. A few mutated oncogenes such as the *EGFR* and *PIK3CA* have been reported but targeting these alterations has yielded limited success in OSCC [8–10].

The overexpression of non-mutated genes has been associated with tumour progression. Genes can be overexpressed as a result of the amplification of genomic loci, loss of expression of negative regulators, or increased transcription due to aberrant enhancer activity. Gene amplification is a frequent genomic alteration in cancers whereby there is an increase in copy number of a sub-chromosomal region containing the gene of interest. Gene amplification can occur due to genomic instability and/or loss of cell cycle control, both of which are hallmarks of carcinogenesis [11–13]. Usually, segments of the genome that are amplified contain many genes and only a select few of these might contribute to carcinogenesis and progression [11–13]. Developing inhibitors for these targets, and companion diagnostics to identify patients suitable for targeted therapy could improve prognosis and alleviate treatment-related morbidity of cancer patients. Interestingly, the chromosomal cytoband 3q22-3q29 is frequently amplified in a wide range of squamous cell carcinomas, including OSCC [14–17].

**Fig. 1:**
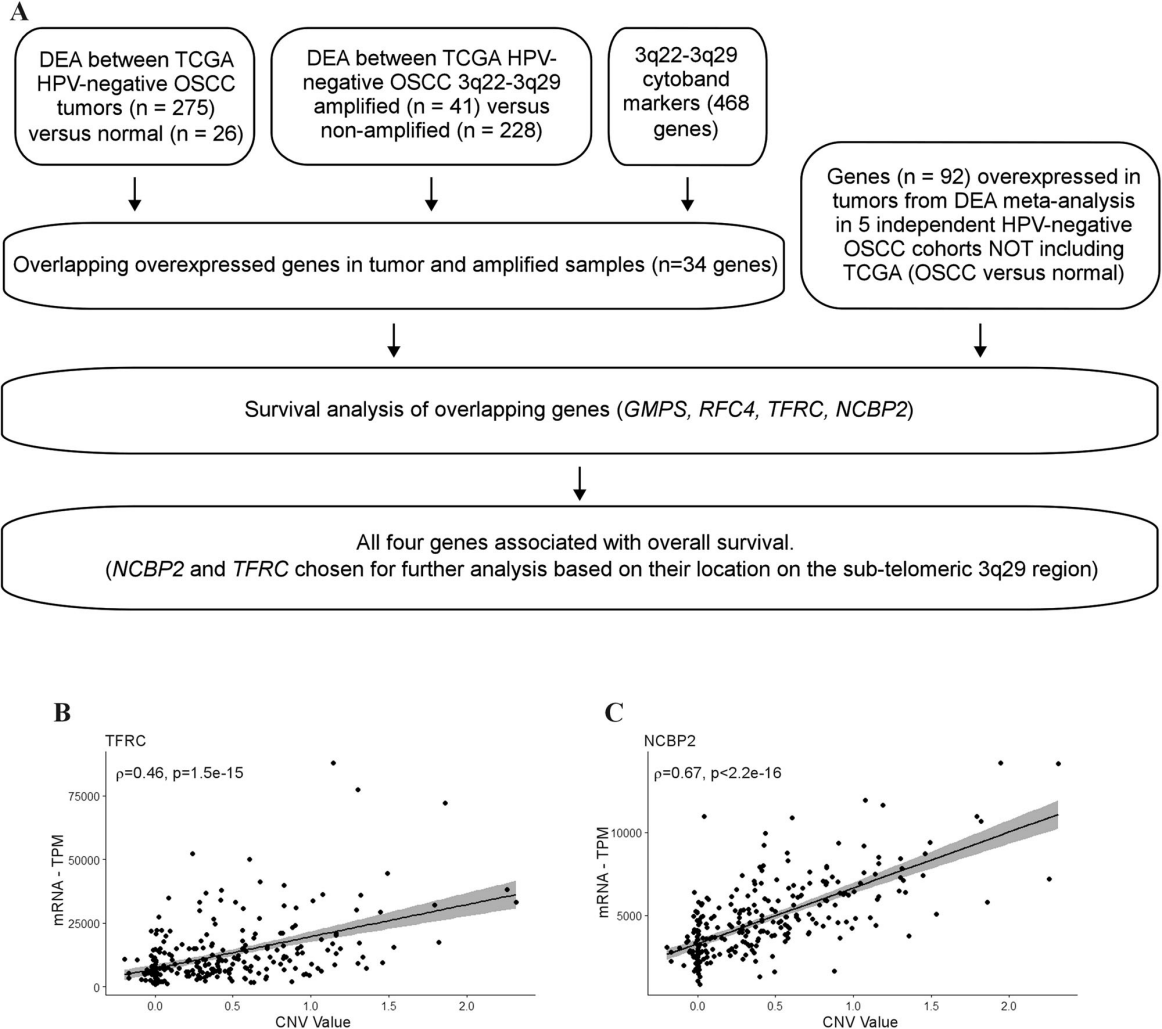
Identification of NCBP2 and TFRC as potential prognostic biomarkers in OSCC. **A** Filtering scheme to identify 3q22-3q29 genes of prognostic significance in HPV-negative OSCC. DEA differential expression analysis. Scatter plots showing the expression of **B** TFRC (Spearman *ρ* = 0.46, *p* < 1.5e−15) and **C** NCBP2 (Spearman *ρ* = 0.67, *p* < 2.2e−16) to be positively correlated to 3q22-3q29 CNV status in TCGA HPV-negative OSCC patients.

Here, we analysed OSCC genomes from The Cancer Genome Atlas (TCGA) to investigate the biological and clinical significance of frequently amplified genes located on the cytobands 3q22-3q29. We devised a simple, yet effective filtering technique (Fig. 1A) to identify genes of clinical relevance (i.e., prognosis) among the 468 genes located on the cytobands 3q22–3q29. Out of four potential hits, two genes, *TFRC* and *NCBP2* that are located on the highly vulnerable telomeric region 3q29, were considered for downstream analyses and validation. High TFRC and NCBP2 protein levels were associated with worse prognosis in OSCC patient-derived tissue microarrays. Since TFRC expression has been previously linked to OSCC cell growth and is considered a potential therapeutic target [18], we investigated the role of NCBP2 in regulating the proliferation, migration and invasion of OSCC cells in culture. Finally, we performed differential expression analysis between top and bottom-quartile *NCBP2*-expressing TCGA OSCC patients to identify potential tumour-promoting factors that are upregulated downstream of *NCBP2*. Our studies have established NCBP2 as a bona fide prognostic marker and potential therapeutic target in OSCC.

## Methods

### Patient cohorts

Demographic, survival, gene expression and copy number data for 282 HPV-negative OSCC patients and 26 normal oral squamous cell samples were obtained from the TCGA data portal. Gene expression data were also obtained for another 263 HPV-negative OSCC patients and 139 normal oral squamous cell samples from five published publicly available datasets [19–23] (Table S1). Gene expression data for normal tissue samples were downloaded from the Genotype-Tissue Expression (GTEx) project. Sample sizes were determined by data availability.

The University of Calgary OSCC cohort consisted of 175 histologically confirmed, surgically resected, treatment-naïve patients diagnosed between 2009 and 2013 (Table 1). The median age was 62.5 years and the median follow-up for the cohort was 5.8 years. Treatment and outcome information is prospectively updated and is current as of January 31, 2022. Patient material and clinical data use to abide by guidelines discussed in the Tri-council Policy Statement for Research with Human Subjects (Canada). This study was performed in accordance with reporting recommendations for tumour marker prognostic studies (REMARK) guidelines [24] and was approved by the Health Research Ethics Board of Alberta (HREBA).

**Table 1:** Demographics of OSCC patients in Ohlson TMA cohort.

Variable *n* (%) unless otherwise indicated	All patients	NCBP2 score 0 or 1	NCBP2 score 2 or 3	*p*-value	TFRC score 0 or 1	TFRC score 2 or 3	*p*-value
Number	175	49	126	–	76	99	
Sex				0.554			0.389
Female	65 (37.1)	16 (32.7)	49 (38.9)		25 (32.9)	40 (40.4)	
Male	110 (62.9)	33 (67.3)	77 (61.1)		51 (67.1)	59 (59.6)	
Median age at diagnosis, years (IQR)	62.5(53.9–74.8)	62.5(51.9–76.7)	62.8(54.6–74.6)	0.774	62.8(53.2–74.0)	62.5(54.6–75.3)	0.682
Pathologic T stage				0.130			0.401
T1/T2	105 (60.0)	26 (53.1)	79 (62.7)		49 (64.5)	56 (56.6)	
T3/T4	62 (35.4)	23 (46.9)	39 (31.0)		24 (31.6)	38 (38.4)	
Missing	8 (4.6)	0 (0.0	8 (6.3)		3 (3.9)	5 (5.1)	
Pathologic N stage				0.217			0.544
Node-negative	63 (36.0)	22 (44,9)	41 (32.5)		28 (36.8)	35 (35.4)	
Node-positive	79 (45.1)	19 (38.8)	60 (47.6)		30 (39.5)	49 (49.5)	
Missing	33 (18.9)	8 (16.3)	25 (19.8)		18 (23.7)	15 (15.2)	
Overall stage				0.803			0.196
Stage I/II	61 (34.9)	19 (38.8)	42 (33.3)		31 (40.8)	30 (30.3)	
Stage III/IV	107 (61.1)	30 (61.2)	77 (61.1)		42 (55.3)	65 (65.6)	
Missing	7 (4.0)	0 (0.0)	7 (5.6)		3 (3.9)	4 (4.0)	
Extracapsular spread				0.337			0.605
Present	27 (15.4)	5 (10.2)	22 (17.5)		10 (13.2)	17 (17.2)	
Absent	148 (84.6)	44 (89.8)	104 (82.5)		66 (86.8)	82 (82.8)	
Differentiation				0.314			0.281
Well	38 (21.7)	11 (22.4)	27 (21.4)		20 (26.3)	18 (18.2)	
Moderate	99 (56.6)	30 (61.2)	69 (54.8)		42 (55.3)	57 (57.6)	
Poor	35 (20.0)	6 (12.2)	29 (23.0)		12 (15.8)	23 (23.2)	
Missing	3 (1.7)	2 (4.1)	1 (0.8)		2 (2.6)	1 (1.0)	
Alcohol consumption history				0.609			0.810
Never drinker	30 (17.1)	10 (20.4)	20 (15.9)		14 (18.4)	16 (16.2)	
Ever drinker	135 (77.1)	36 (73.5)	99 (78.6)		57 (75.0)	78 (78.8)	
Missing	10 (5.7)	3 (6.1)	7 (5.6)		5 (6.6)	5 (5.1)	
Smoking history				0.647			0.354
Never smoker	48 (26.3)	11 (22.4)	35 (27.8)		23 (30.3)	23 (23.2)	
Ever smoker	128 (73.1)	37 (75.5)	91 (72.2)		52 (68.4)	76 (76.7)	
Missing	1 (0.6)	1 (2.0)	0 (0)		1 (1.3)	0 (0)	
Median pack-years smoked (IQR)	20 (0–35)	25 (1.7–38.5)	18 (0–30)	0.224	13.5 (0–30)	20 (0.2–40.0)	**0.015**

*P*-values were calculated with the Mann–Whitney *U* test for continuous variables and the *χ*^2^ test or Fisher’s exact test for categorical variables, as appropriate.

### Determination of 3q22-3q29 amplification status

Genomic Identification of Significant Targets in Cancer (GISTIC) level 2 data containing amplification statuses for notable amplifications of each TCGA-OSCC sample was downloaded from the Broad Institute’s Genome Data Analysis Center (GDAC) Firehose. Specifically, the file analysed was “all_lesions.conf_99.txt”. The 3q22-3q29 amplification status came from peak 3 of this file (3q26.33, region limits: chr3:131468786-198022430; 3q22-3q29). 282 samples were considered for differential expression analyses between 3q22-3q29 amplified (*n* = 41 samples) and non-amplified (*n* = 236 samples) TCGA HPV-negative OSCC samples. Amplification data was unavailable for 7 samples. Markers for this cytoband region were determined by filtering the “all_data_by_genes.txt” GISTIC2 output file (downloaded from GDAC firehose) for 3q22-29 (*n* = 468 genes).

### Differential expression analysis (DEA)

Level III mRNA-sequencing data (raw counts) was used to perform DEA using the DESeq2 Bioconductor package [25]. DEA was performed between primary TCGA HPV-negative OSCC tumour versus normal tissue samples, and between 3q22-3q29 amplified versus non-amplified primary tumour samples. Thresholds for differential expression were set at an absolute fold change cut-off of 1.5 and false discovery rate (FDR) of 0.1% (adjusted *p* < 0.001). Differentially expressed genes were filtered for genes located on the 3q22-3q29 cytoband. Filtering steps are described in the flow chart shown in the appended Fig. 1A. Briefly, the genes overexpressed in 3q22-3q29 amplified tumour samples overlapped with the genes overexpressed on OSCC compared to matched normal samples. We also overlapped these commonly overexpressed genes with those overexpressed in OSCC samples from five independent HPV-negative OSCC cohorts containing both OSCC and matched normal oral cavity squamous epithelium. The final set of genes was subjected to univariate cox proportional hazard analysis with overall survival as an outcome and significant hits were further analysed.

DEA was performed between top quartile *NCBP2* and bottom quartile *NCBP2* expressing OSCC patients in the TCGA. Thresholds for differential expression were set at an absolute fold change cut-off of 1.5 and FDR of 5% (adjusted *p* < 0.05). An extensive literature review was performed to evaluate the roles (if any) of the DE genes in regulating OSCC tumorigenesis and progression.

### TCGA OSCC promoter DNA methylation and mRNA gene expression analysis

R studio (version 4.2.0) and R package TCGAbiolinks (version 2.24.3) were used to analyse the TCGA HPV-negative OSCC samples. TCGAbiolinks was used to download DNA methylation beta values (Illumina Human Methylation 450 array) and transcriptome profiling mRNAseq counts for all samples. DNA methylation beta values and mRNAseq counts were filtered for four genes (*NCBP2, TFRC, RFC4*, and *GMPS*). mRNAseq raw counts were normalized to TPM values. CpG promoter probes for all four genes were identified and filtered for by genomic base pair position. *R* function cor.test was used to calculate spearman correlation values between CpG promoter probes and log_2_TPM (mRNA) counts. Firstly, individual CpG promoter probes were correlated with log_2_TPM (mRNA) counts, *ρ* and *p*-values are reported. Secondly, scatter plots of mean promoter beta value versus log_2_TPM (mRNA) counts are shown for the four genes.

### Tissue microarray (TMA) construction

Haematoxylin–eosin (H&E)-stained slides were reviewed by the study pathologist (DI) to select formalin-fixed paraffin-embedded (FFPE) tissue blocks with sufficient tumour content. Three 0.6 mm cores were randomly sampled for each patient from tumour-bearing areas of the FFPE tissue blocks using a beecher manual tissue microarrayer (Beecher Instruments Inc., WI, USA) and arrayed on TMA blocks. Tissue represented ~70 patients (in triplicate) on each TMA block. Normal oral cavity squamous epithelium tissue cores were also included. TMA slides were prepared using 4 μm-thick sections from the TMA block.

### Immuno-histochemistry (IHC)

TMA slides were deparaffinized and rehydrated before performing heat-induced epitope retrieval. Endogenous peroxidase activity was quenched with a peroxidase block, and slides were blocked and permeabilized using rodent block with 0.2% Triton X. Slides were incubated for one hour with anti-NCBP2 (abcam ab91560) or anti-TFRC antibodies (abcam ab84036) for one hour followed by an HRP-conjugated mouse anti-rabbit secondary antibody (Dako EnVison+ kit, K4065), and finally for 2 min with 3,3′-diaminobenzidine tetrahydrochloride (DAB) to visualize bound antibodies. Slides were counterstained with hematoxylin, dehydrated, and mounted. Multiple antibody concentrations for NCBP2 (1:500, 1:1000, 1:2000 and 1:2500) and TFRC (1:100, 1:500, 1:1000, 1:1500, 1:2000, 1:2500 and 1:5000) were used to optimize staining for both proteins in control mouse liver and normal oral cavity squamous epithelium core samples (Fig. S1). The study pathologist (DI) assessed stained slides to select the optimal antibody concentration that would permit for scoring of differential protein expression levels within TMA cores. After staining optimization, final primary antibody concentrations of 1:2000 for NCBP2 and 1:1500 TFRC were selected. TMAs were scored by the pathologist (DI) with a score of 0 indicating negative staining, and scores of 1, 2 or 3 indicating positive staining of increasing intensity for each protein. Disagreements between cores from the same case were resolved by taking the maximum score for that patient.

### Statistical and survival analysis

Univariate Cox proportional hazards regression was used to determine the association between biomarkers and clinical covariates and OS, DSS, or PFI. Covariates showing significant associations in univariate analysis were adjusted with *p*-value correction or multivariate models depending on the analysis. Kaplan–Meier survival curves were used to visualize differences in survival between groups. For Kaplan–Meier curves with continuous variables, a cut-point determined by the method outlined by Contal and O’Quigley [26] was utilized. The protein expression status of University of Calgary OSCC patients was dichotomized based on low (0/1) and high (2/3) NCBP2 and TFRC protein levels for Kaplan–Meier curves.

Demographic differences by gene and protein expression levels were assessed using the Mann-Whitney *U* test for continuous variables and Pearson’s *χ*^2^ test for categorical variables. Spearman’s *ρ* was calculated for the correlation between *NCBP2* and *TFRC* mRNA and protein expression. *NCBP2* and *TFRC* mRNA expression was compared between tumour and matched normal tissue using the Wilcoxon signed-rank test. Unpaired *t*-test was performed for the in vitro assays. All statistical analyses were performed using GraphPad Prism (GraphPad, California) or R version 3.6.1.

### Immunoblotting analysis

Protein lysates were isolated from the UMSCC29 and CAL33 cell lines treated with a control siRNA or a pool of siRNA targeting NCBP2 (NCBP2i), using TNTE (50 mM Tris, 150 mM NaCl, and 1 mM EDTA) buffer containing 1% Triton X-100 along with protease and phosphatase inhibitors, and 0.1% SDS. Cell extracts were collected in Eppendorf tubes and centrifuged at 14,000×*g* for 10 min at 4 °C. Equivalent protein amounts of lysates were resolved by SDS–PAGE followed by transfer to nitrocellulose membranes. Specific proteins on membranes were incubated overnight with primary antibodies targeting NCBP2 (abcam ab91560 rabbit anti-human NCBP2, 1:4000), and actin (Santa Cruz sc-47778, 1:2000) in 3% BSA. Membranes were then incubated with HRP-conjugated goat anti-mouse or donkey anti-rabbit IgG (Bio-Rad Laboratories, 1:10,000 in 5% skim milk) as secondary antibodies for 1 h at room temperature, followed by incubation in enhanced chemiluminescence (ECL) (Millipore) reagent and light signal detection using a Chemidoc® Touch Imager (Bio-Rad Laboratories).

### Cell proliferation assay

5 × 10^5^ UMSCC29 and CAL33 cells were seeded in each well of a 12-well tissue culture plate and treated either with control RNAi or NCBP2i for 24 h after which 5000 cells each were sub-cultured in triplicate in a 96-well tissue culture plate overnight. BrDU reagent was added, and proliferation was measured using a BrDU Cell Proliferation ELISA kit (Abcam, ab126556). The mean ± SEM of relative proliferating cells of the independent experiments is plotted on the *y*-axis versus the experimental conditions on the *x*-axis of a bar graph.

### In vitro scratch assay

5 × 10^5^ UMSCC29 and CAL33 cells were seeded in each well of a 12-well tissue culture plate and treated either with control RNAi or NCBP2i for 48 h and grown to near confluency in complete growth medium and then 24 h serum starved by incubating with 0.2% FBS-containing cell culture medium in a 5% CO_2_ humidified incubator at 37 °C. Using a 200 μL pipette tip, a scratch was introduced along the midline of the serum-starved cell monolayers, followed by a PBS wash to remove floating cells, and incubation of the cells with 0.2% FBS-containing medium for 30 or 48 h, for UMSCC29 and CAL33 cells, respectively, in a 5% CO_2_ humidified incubator at 37 °C. Scratch closure in each well was followed by imaging the scratch and surrounding cells in each well at ×10 objective of a DIC microscope (Olympus CKX53) coupled to a digital camera at times 0 and 30 or 48 h after initiating the scratch. Five images were captured along the vertical axis of the scratch for each experimental condition. The width of each scratch was measured at three different positions per image for a total of 15 measurements using ImageJ (National Institutes of Health, USA), and then averaged per experimental condition. The width average at the endpoint was subtracted from the width average at 0 h and expressed relative to that at 0 h width for each experimental condition to obtain scratch closure and expressed as percent scratch closure. The mean ± SEM of relative scratch closure of the independent experiments is plotted on the *y*-axis versus the experimental conditions on the *x*-axis of a bar graph.

### In vitro transwell invasion assays

Overnight 0.2% FBS-containing media, i.e. serum-starved, control RNAi or NCBP2i treated, UMSCC29 and CAL33 cells were used for the transwell invasion using polycarbonate filters (24-well inserts, pore size 8 μm; BD Biosciences, Canada). Prior to the addition of cells, each insert was placed within a well of a 24-well tissue culture plate and equilibrated with 0.5 mL serum-free DMEM, added both to the upper and lower chambers at 37 °C for 2 h. The equilibration media was then gently removed and the upper chamber surface of the insert was coated with 50 μL of 3% Matrigel and allowed to solidify at 37 °C for 1 h. 2 × 10^5^ serum-starved OSCC cells were resuspended in 0.5 mL of serum-free DMEM and added to the upper Matrigel-coated chamber. 500 μL complete growth medium was added to the lower chamber. Cells were allowed to invade the matrix overnight at 37 °C after which non-adherent cells were removed by PBS washing of cell layers on the upper chamber three times. During the second wash, a cotton tip applicator was used to gently scrape away the adherent cells on the upper surface of the membrane. Invading cells were fixed by immersing the transwell inserts in 100% methanol for 20 min at −20 °C, followed by staining with 0.5% crystal violet dye (EMD Millipore, Canada) for 1 h at room temperature. Six randomly chosen fields of each stained membrane were imaged at ×10 objective of a DIC microscope (Olympus CKX53) coupled to a digital camera. Crystal violet-stained cells in each field were counted using a handheld counter and an average count of cells for the six fields per condition was obtained. Each experiment was repeated at least three independent times, and invading cell counts at each experimental condition were expressed relative to the respective control RNAi-treated condition. The mean ± SEM of relative invading cells of the independent experiments is plotted on the *y*-axis versus the experimental conditions on the *x*-axis of a bar graph.

## Results

### The expression of NCBP2 and TFRC genes is associated with clinical outcomes

To identify putative oncogenic drivers among the 468 genes on 3q22–3q29, we performed a series of DEA and survival analyses (Fig. 1A). First, we performed DEA between 3q22-3q29 amplified (41) vs. non-amplified (228) TCGA-OSCC samples and DEA between TCGA-OSCC tumours (275) vs. normal samples (26). Then we performed a DE meta-analysis between OSCC and normal samples from six different datasets (Table S1), including TCGA [19–23]. After filtering the genes to those that were common in all these DE analyses and to the 468 genes on 3q22-3q29, we observed that the overexpression of four genes (*GMPS*, *RFC4*, *NCBP2* and *TFRC*) were directly associated with 3q22-3q29 amplification (Fig. S2). We also investigated if the expression of the four genes was correlated with the methylation of their respective promoters, another mechanism by which gene expression is altered in malignant tissue [27]. However, we did not find a significant correlation between beta-values derived from TCGA Illumina Infinium Human Methylation 450K array data and the expression levels of *GMPS*, *RFC4*, *NCBP2* and *TFRC* genes (Fig. S3 and Table S2) in OSCC.

To better understand the clinical relevance of the four genes driven by 3q22-3q29 amplification, we performed survival analysis with OS as endpoint in HPV-negative OSCC samples. 3q22-3q29 amplification itself was not associated with OS in HPV-negative OSCC patients (Fig. S4). However, increased expression of all four genes was associated with worse OS in HPV-negative OSCC samples (Cox proportional hazard ratio > 1; *p*-value < 0.05). Several studies have described that telomeric aberrations play a critical role in tumourigenesis [28]. Thus, we selected *NCBP2* and *TFRC*, both genes on the telomeric cytoband 3q29, for further analyses. In concordance with the DE analyses described above, we found the gene expression of *TFRC* and *NCBP2* faithfully tracked 3q22-3q29 amplification status in TCGA HPV-negative OSCC samples (Fig. 1B, C).

**Fig. 2:**
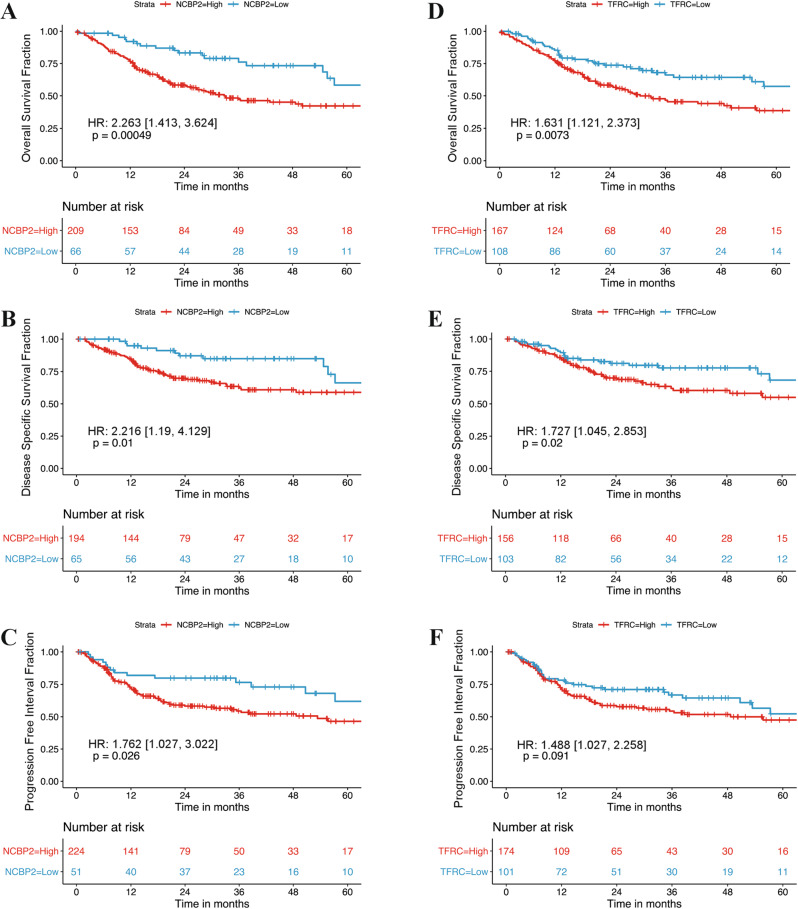
Kaplan–Meier survival curves for TCGA OSCC patients dichotomized by optimized high vs. low NCBP2 and TFRC expression. **A**–**C** Patients with high NCBP2 expression had significantly poorer OS, DSS, and PFI. **D**–**F** Patients with high TFRC expression had significantly poorer OS and DSS, but not PFI. Hazard ratios and *p*-values quoted are from univariate coxph models, values in square brackets indicate 95% confidence intervals.

Upon performing univariate Cox proportional hazards (Coxph) analysis in TCGA HPV-negative OSCC patients, high *NCBP2* expression was associated with significantly worse OS (hazard ratio [HR] = 1.7009 [95% CI: 1.171–2.47], *p* = 0.00527), but not DSS (HR = 1.3631 [95% CI: 0.8386–2.216], *p* = 0.211), or PFI (HR = 1.1368 [95% CI: 0.7691–1.68], *p* = 0.52). High *TFRC* expression was also associated with significantly worse OS (HR = 1.3152 [95% CI: 1.069–1.618], *p* = 0.0094)], but not DSS [HR = 1.1944 [95% CI: 0.9097–1.568], *p* = 0.201), or PFI [HR = 1.09919 [95% CI: 0.8786–1.375], *p* = 0.408). Kaplan–Meier (KM) visualization with optimized cut-points showed that high *NCBP2* expression was associated with worse OS, DSS, and PFI (Fig. 2A–C). Similarly, patients with high *TFRC* expression were associated with worse OS and DSS, but not PFI (Fig. 2D–F). Although the continuous mRNA levels of *NCBP2* or *TFRC* are not significantly associated with DSS and PFI in univariate Coxph analysis, the KM visualizations indicate that these biomarkers may still retain value for the risk stratification in OSCC.

Clinico-pathological characteristics of the HPV-negative TCGA OSCC patients stratified by median *TFRC* and *NCBP2* mRNA expression are presented in Table 2. Patients with high *NCBP2* expression were more likely to be male, African-American and Asian by race and present with higher histologic grade. Patients with high *TFRC* expression were more likely to be male and present with a higher pathologic stage. There were no significant group differences observed for the median age of diagnosis, subsite, alcohol consumption history, smoking history, and nodal extracapsular spread. We also observed a significant correlation between *NCBP2* and *TFRC* expression in OSCC patients (Spearman *ρ* = 0.68, *p* < 2.2e−16; Fig. S5), which is expected due to their co-location on the same chromosomal cytoband that is amplified.

**Table 2:** Demographics of OSCC patients in TCGA cohort.

Variable *n* (%) unless otherwise indicated	All patients	NCBP2 expression below median	NCBP2 expression above median	*p*-value	TFRC expression below median	TFRC expression above median	*p*-value
Number	275	137	138	–	137	138	–
Sex				**0.010**			**0.020**
Male	181 (65.8)	80 (58.4)	101 (73.2)		81 (59.1)	100 (72.5)	
Female	94 (34.2)	57 (41.6)	37 (26.8)		56 (40.9)	38 (27.5)	
Median age at diagnosis, years (IQR)	62 (53–71)	63 (53–73)	61 (54–69)	0.689	61 (52–71)	63 (55–71)	0.343
Race				**0.003**			0.052
White	237 (86.2)	128 (93.4)	109 (79.0)		125 (91.2)	112 (81.2)	
African-American	19 (6.9)	4 (2.9)	15 (10.9)		4 (2.9)	15 (10.9)	
Asian	9 (3.3)	1 (0.7)	8 (5.8)		4 (2.9)	5 (3.6)	
American Indian	1 (0.4)	0 (0)	1 (0.7)		0 (0)	1 (0.7)	
Unknown	9 (3.3)	4 (2.9)	5 (3.6)		4 (2.9)	5 (3.6)	
Oral cavity				0.524			0.127
Oral tongue	112 (40.7)	55 (40.2)	57 (41.3)		67 (48.9)	45 (32.6)	
Oral cavity	65 (23.6)	38 (27.7)	27 (19.6)		30 (21.9)	35 (25.4)	
Floor of mouth	57 (20.7)	25 (18.3)	32 (23.2)		24 (17.5)	33 (23.9)	
Buccal mucosa	22 (8.0)	9 (6.6)	13 (9.4)		8 (5.8)	14 (10.1)	
Alveolar ridge	13 (4.7)	6 (4.4)	7 (5.1)		5 (3.7)	8 (5.8)	
Hard palate	6 (2.2)	4 (2.9)	1 (1.5)		3 (2.2)	3 (2.2)	
Pathologic stage				0.121			**0.001**
Stage I/II	65 (25.5)	37 (29.8)	28 (21.4)		43 (35.0)	22 (16.7)	
Stage III/IV	190 (74.5)	87 (70.2)	103 (78.6)		80 (65.0)	110 (83.3)	
Extracapsular spread				0.236			0.073
Present	54 (19.6)	23 (16.8)	31 (22.5))		21 (15.3)	33 (23.9)	
Absent	221 (80.4)	114 (83.2)	107 (77.5)		116 (84.7)	105 (76.1)	
Histological grade				**0.015**			0.086
Grade 1	49 (17.8)	33 (24.1)	16 (11.6)		32 (23.4)	17 (12.3)	
Grade 2	168 (61.1)	74 (54.0)	94 (68.1)		75 (54.7)	93 (67.4)	
Grade 3	54 (19.6)	27 (19.7)	27 (19.6)		29 (21.2)	25 (18.1)	
Alcohol consumption history				0.340			0.314
0 drinks daily	37 (32.2)	20 (37.7)	17 (27.4)		20 (35.1)	17 (29.3)	
1–3 drinks daily	40 (34.8)	15 (28.3)	25 (40.3)		22 (38.6)	18 (31.0)	
>3 drinks daily	38 (33.0)	18 (34.0)	20 (32.3)		15 (26.3)	23 (39.7)	
Median pack-years smoked (IQR)	40 (25–54)	37 (20–51)	40 (25–60)	0.101	37 (25–54)	40 (25–60)	0.259

*p*-values calculated with the Mann–Whitney *U* test for continuous variables and the *χ*^2^ test or Fisher’s exact test for categorical variables, as appropriate.

Significant *p*-values are bolded.

### NCBP2 and TFRC are potential therapeutic targets

**Fig. 3:**
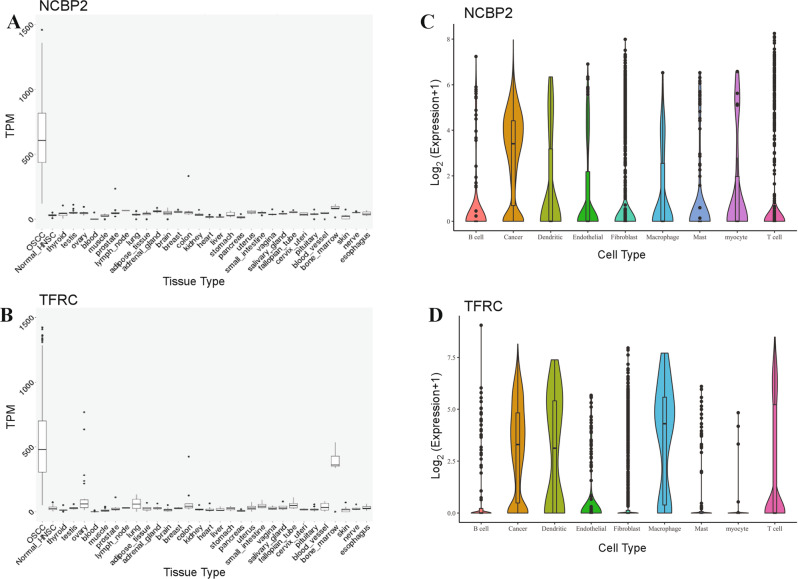
Altered expression of NCBP2 and TFRC in OSCC cells. mRNA expression of NCBP2 (**A**) and TFRC (**B**) in OSCC relative to various normal tissues shows higher expression in OSCC as compared to most normal human tissue types. Transcripts per million (TPM) data was obtained from TCGA and Genotype-Tissue Expression (GTEx) project. Single-cell mRNA expression of NCBP2 (**C**) and TFRC (**D**) in 18 head and neck squamous cell carcinoma as available from Puram et al. [29] showing high expression in OSCC cells as opposed to other cells in the tumour microenvironment.

Since both increased *NCBP2* and *TFRC* expression are associated with worse survival in OSCC, these genes could potentially be targeted for therapeutic benefit. However, systemic administered targeted therapies would also suppress the expression of these genes in other high-expressing normal tissues, potentially resulting in adverse side effects. Analysis of GTEx and TCGA expression data revealed that *NCBP2* and *TFRC* expression was negligible across all normal tissues evaluated (except for *TFRC* expression in the bone marrow) and was significantly higher in OSCC samples (Fig. 3A, B). Analysis of single-cell RNAseq data from a study published by Puram et al. [29] also shows *NCBP2* expression to be significantly higher in OSCC cells compared to other cell types in the tumour microenvironment, while *TFRC* expression was observed in tumour cells, dendritic cells, and macrophages (Fig. 3C, D). Thus, this provides a therapeutic window for targeting NCBP2 and to a lesser extent TFRC in OSCC patients with 3q22-29 amplification and *NCBP2*/*TFRC* overexpression.

### TFRC and NCBP2 protein abundance is associated with demographic differences and patient survival

**Fig. 4:**
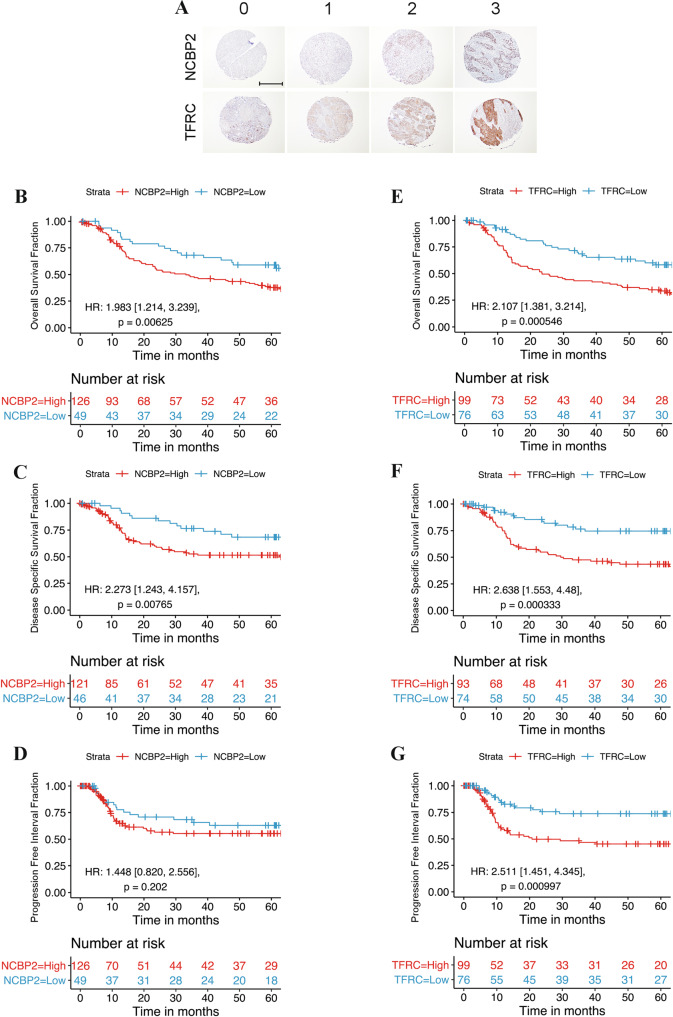
Kaplan–Meier survival curves for Ohlson OSCC patients dichotomized by optimized high vs. low NCBP2 and TFRC protein abundance. **A** Representative TMA slides for each NCBP2 and TFRC staining score established by a trained pathologist. A score of 0 indicates no antibody staining, and scores of 1, 2 and 3 indicate positive stains of increasing intensity. All positive stains show specific staining primarily localized to the nucleus for NCBP2 and to the cytoplasm and membrane for TFRC, corresponding to their cellular localizations. Kaplan–Meier survival curves by high (2/3) vs. low (0/1) NCBP2 and TFRC expression reveal patients with high NCBP2 expression had significantly poorer OS (**B**), DSS (**C**), but not PFI (**D**). Patients with high TFRC expression had significantly poorer OS (**E**), DSS (**F**), and PFI (**G**). Hazard ratios and *p*-values quoted are from univariate coxph models, values in square brackets indicate 95% confidence intervals. Scale bars indicate 50 μm.

To mitigate the often-observed poor correlation between mRNA and protein levels and the limitations inherent in TCGA clinical data, we characterized NCBP2 and TFRC protein expression using IHC in TMAs constructed from a retrospective cohort of OSCC patients at the University of Calgary (Fig. 4A). NCBP2 and TFRC specific IHC conditions were optimized using normal mouse and human tissue samples (Fig. S1). Of the 183 total patients in the TMA cohort, 8 (4.3%) could not be assayed for both genes and were removed from further analysis, leaving 175 analysed patients. NCBP2 and TFRC proteins were primarily expressed in the nuclear and cytoplasmic compartments, respectively, and expression of both proteins was primarily restricted to squamous cell carcinoma cells in the tumour microenvironment. Table 2 describes the demographics of the University of Calgary OSCC cohort stratified by NCBP2 and TFRC protein levels. Patients with high TFRC protein expression had smoked more pack-years (*p* = 0.015). NCBP2 and TFRC protein levels were not correlated in OSCC patients (Spearman’s correlation (*ρ*) = 0.082, *p* = 0.283; Table S3) unlike the mRNA levels, suggesting that protein expression may be influenced by post-transcriptional and post-translational modifications.

We further sought to determine the clinical impact of NCBP2 and TFRC protein levels in OSCC patients by performing survival analyses with OS, DSS and PFI as end-points. Comparisons of OS, DSS and PFI were first conducted based on the continuous TFRC and NCBP2 protein scores. In univariate Coxph analysis, patients with higher NCBP2 or TFRC expression were associated with significantly worse OS, DSS, and PFI (Table 3).

**Table 3:** Cox regression for survival conditions among OSCC patients in the Ohlson TMA cohort.

Variable	Univariate analysis				Multivariate analysis			
	*p*-value	Hazard ratio (HR)	95% CI for HR		*p*-value	Hazard ratio (HR)	95% CI for HR	
			Lower	Upper			Lower	Upper
*Overall survival*								
Age at diagnosis	3.09*10^**−6**^	1.035	1.020	1.050	4.75*10^**−9**^	1.048	1.032	1.065
Clinical Stage I/II vs. III/IV	**0.002**	2.143	1.336	3.438	0.243	1.352	0.815	2.243
Extracapsular spread (present)	6.2*10^**−6**^	3.038	1.876	4.919	7.03*10^**−9**^	5.365	3.038	9.473
TFRC protein score	**0.002**	1.464	1.157	1.852	5.39*10^**−4**^	1.561	1.213	2.009
NCBP2 protein score	**0.005**	1.436	1.117	1.846	**0.016**	1.381	1.062	1.796
*Disease-specific survival*								
Median age	**0.001**	1.03	1.012	1.048	1.95*10^**−5**^	1.050	1.027	1.073
Pathological stage Stage I/II vs. III/IV	1.04*10^**−4**^	4.045	1.997	8.192	**0.012**	2.558	1.229	5.324
Extracapsular spread (present)	4.08*10^**−5**^	3.185	1.831	5.54	1.47*10^**−6**^	5.401	2.719	10.729
TFRC protein score	4.49*10^**−4**^	1.677	1.256	2.239	6.14*10^**−4**^	1.717	1.260	2.339
NCBP2 protein score	**0.003**	1.589	1.172	2.154	**0.012**	1.486	1.092	2.023
*Progression-free interval*								
Median age	0.076	1.016	0.998	1.035	**0.028**	1.023	1.002	1.044
Clinical Stage I/II vs. III/IV	**0.015**	2.062	1.154	3.684	0.260	1.429	0.768	2.661
Extracapsular spread (present)	**0.002**	2.570	1.432	4.614	**0.002**	2.862	1.458	5.622
TFRC protein score	**0.009**	1.469	1.100	1.963	**0.016**	1.438	1.069	1.935
NCBP2 protein score	**0.040**	1.392	1.015	1.910	**0.049**	1.368	1.001	1.871

Significant *p*-values are bolded.

Kaplan–Meier visualizations were then performed to further assess the prognostic value of these protein biomarkers. TFRC and NCBP2 protein scores were dichotomized into high (+2/+3) or low (0/+1) groups. Patients with high NCBP2 protein expression had significantly worse OS and DSS, but not PFI (Fig. 4B–D). Patients with high TFRC protein expression also had significantly worse OS, DSS, and PFI (Fig. 4E–G).

### NCBP2 and TFRC protein expression is associated with clinical outcomes on multivariate analysis

Since association with survival outcomes may be confounded by other clinical variables, we performed multivariate Coxph analysis to control for relevant clinical covariates. In univariate Coxph, pathological stage (I/II vs. III/IV), extracapsular spread, and the continuous TFRC and NCBP2 protein scores were each significantly associated with worse OS, DSS, and PFI. Age at diagnosis was significantly associated with OS and DSS, but not PFI. A multivariate Cox model was constructed using these covariates, which found age, extracapsular spread, TFRC protein score and NCBP2 protein score to be associated with worse OS. Age, clinical stage, extracapsular spread, TFRC protein score and NCBP2 protein score were all associated with worse DSS in multivariate Cox analysis. Age, extracapsular spread, TFRC protein score and NCBP2 protein score were all associated with worse PFI on multivariate Cox analysis (Table 3).

### NCBP2 depletion suppresses OSCC cell migration, invasion, and proliferation

**Fig. 5:**
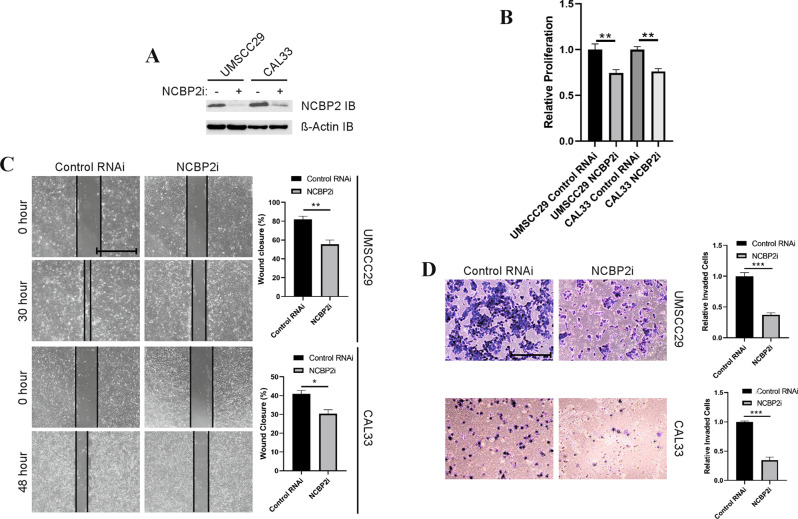
Association of NCBP2 depletion in OSCC cell lines with oncogenic progression. **A** Immunoblot showing reduction in NCBP2 levels after siRNA (NCBP2i)-mediated knockdown in UMSCC29 and CAL33 cell lines. β-actin immunoblot was performed as a control. Reduction in endogenous NCBP2 levels by NCBP2i led to reduced OSCC cell proliferation (**B**), migration (**C**), and invasion (**D**) of both UMSCC29 and CAL33 cells. Scale bars indicate 500 μm. Statistical significance (unpaired *t*-test): *p < 0.05, **p < 0.01, and ***p < 0.001.

TFRC protein has been shown to regulate the progression of several squamous epithelial tumours [18, 30, 31], however, no functional analysis has been performed on the ability of NCBP2 to regulate tumour progression. Therefore, we evaluated the functional relevance of NCBP2 on OSCC progression using appropriate cell line-based analyses. We depleted *NCBP2* expression in two OSCC cell lines UMSCC29 and CAL33 using a siRNA pool (NCBP2i). Immunoblotting analysis showed a marked reduction in NCBP2 protein abundance in NCBP2i-treated cells as compared to those treated with scrambled siRNA (Fig. 5A). Using a BrDU incorporation assay we observed that depletion of endogenous NCBP2 led to reduced cell proliferation of both the cell lines (Fig. 5B). In vitro scratch assays were performed to test the effect of NCBP2 knockdown on the migratory behaviour of OSCC cells. We found that NCBP2 depletion significantly reduced the speed of scratch closure compared to control in both UMSCC29 and CAL33 cells (Fig. 5C). In addition to migration, invasion plays an important role in the ability of cancer cells to move to sites outside the primary tumour site and initiate metastasis. The effect of NCBP2 knockdown on the invasive ability of OSCC cells was tested using transwell Matrigel^TM^ assay. NCBP2i significantly reduced the relative number of invading cells as compared to the control in both cell lines (Fig. 5D).

### NCBP2 promotes the expression of several genes involved in tumour progression in OSCC

**Fig. 6:**
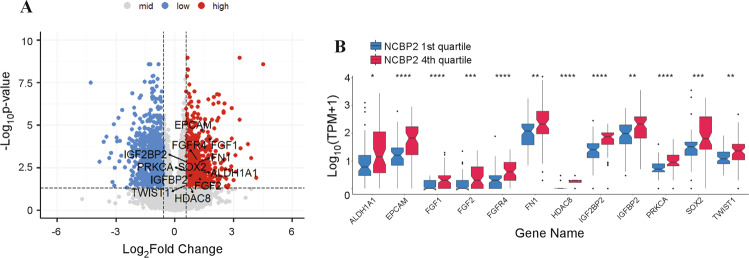
Upregulated expression of specific genes in NCBP2-amplified TCGA OSCC patient samples. **A** Volcano plot showing the differentially expressed genes in *NCBP2* 4th quartile vs. 1st quartile expressing HPV-negative OSCC tumours with well-established oncogenic drivers highlighted. **B** Boxplots showing the significantly elevated expression of 12 oncogenic drivers of OSCC in NCBP2 high-expressing tumours compared to low-expressing. Statistical significance (Wilcoxon signed rank test): *p < 0.05, **p < 0.01, ***p < 0.001, and ****p < 0.0001.

Since our studies indicate that NCBP2 is a novel tumour-promoting gene and very little is known about the downstream effector genes regulated by NCBP2, we performed DEA between top (*n* = 68) vs. bottom quartile (*n* = 69) *NCBP2* expressing TCGA-OSCC samples. We filtered the resulting gene list based on an absolute fold change >1.5 and FDR of 5% (*p* < 0.05) (Fig. 6A). We then performed an extensive literature review to shortlist 12 genes with well-studied oncogenic roles in OSCC and potential targeted therapies available or in development targeting these genes (Table 4, Fig. 6B). These results corroborate the tumour-promoting effects of NCBP2 observed in our functional studies and provide important clues to how downstream NCBP2 signalling may lead to a more aggressive tumour abetting phenotype and how such signalling may be effectively targeted for therapeutic benefit.

**Table 4:** The fold change, adjusted *p*-value, reported role in OSCC and therapeutics that are known to target genes upregulated in NCBP2-overexpressing patients.

Gene name	Protein coded	Fold change	Adjusted *p*-value	Role in OSCC	Known therapeutic interventions
ALDH1A1	Aldehyde dehydrogenase 1A1	2.68	5.63E−03	High ALDH1A1 expression was associated with higher TNM tumour stage and high nodal stage and high mortality rate in OSCC [43]	Disulfiram (DSF)–copper (Cu) complex [44].
EPCAM	Epithelial cell adhesion molecule	2.75	2.27E−05	EpCAM regulates cyclin D1 expression and localization in OSCC cells under anchorage-independent conditions [45]. Overexpression of EpCAM was associated significantly with OSCC tumour size, histological grade, local recurrence of tumour and patient survival [46, 47] EpCAM reduction decreased the invasion potential and proliferation activity of OSCC cells [47]	Several monoclonal antibodies [48].
FGF1	Fibroblast growth factor 1	3.39	5.26E−05	FGF1 promotes EMT and thus invasion and metastasis in OSCC [49, 50]	FGF signalling axis targeting small-molecule receptor TKIs (non-selective, selective and covalent), monoclonal antibodies, FGF ligand traps and DNA/RNA aptamers [51]
FGF2	Fibroblast growth factor 2	2.30	1.30E−02	There is a significant association of FGF-2 expression with malignant transformation of OSCC and high-grade tumours and correlated with the presence of metastasis and adverse outcomes [52, 53]	
FGFR4	Fibroblast growth factor receptor 4	2.20	1.08E−03	FGFR4 is frequently overexpressed in OSCC [54, 55]	
FN1	Fibronectin 1	2.29	1.55E−03	FN1 is upregulated and correlates to poor prognosis in OSCC [56] and promotes the proliferation, invasion and migration of OSCC cells [57]	Expression of fibronectin 1 or its binding to its partners are attractive targets for novel drug development [58]
HDAC8	Histone deacetylase 8	1.76	4.91E−02	HDAC8 is overexpressed in OSCC tissues and cell lines. HDAC8 silencing enhanced apoptosis, suppressed cell growth and metastasis in OSCC cells [59, 60]	Several HDAC inhibitors have shown promise in suppressing tumour progression [61]. HDAC8-specific inhibitor PCI-34051 with a >200-fold selectivity over other HDAC isoforms has shown promise in preclinical models of HCC [62]
IGF2BP2	Insulin growth factor 2 binding protein 2	1.58	1.55E−03	IGF2BP2 was highly expressed in OSCC and significantly correlated with poor overall survival of OSCC patients. Apoptosis-, tumour-, and immune-related pathways were significantly enriched in samples with high IGF2BP2 expression. IGF2BP2 co-expressed genes indicated that these genes are mainly associated with immunity/inflammation and tumorigenesis. In addition, IGF2BP2 and its co-expressed genes are associated with TICs [63, 64]	Very recently, a few targeted therapies are being evaluated for IGF2BP2 (35023719, 35915142)
IGFBP2	Insulin growth factor binding protein 2	1.88	7.46E−03	IGFBP-2 is strongly correlated with oral cancer metastasis and progression [65]	Antisense oligonucleotide or neutralizing antibodies have been developed to target IGFBP2 [66]
PRKCA	Protein kinase C alpha	1.53	4.97E−03	PRKCA overexpression is enriched in young OSCC patients and is associated with poor prognosis [67]	There is a lack of isozyme-specific PKC inhibitors. However, a few are being developed [68]
SOX2	SRY-box 2	2.29	4.38E−03	SOX2 expression is an independent predictor of oral cancer risk in patients with oral leukoplakia [69]. SOX2 promotes tumour aggressiveness and epithelial-mesenchymal transition in OSCC and associated with LNM [70, 71]	Very few SOX2 targeting therapeutic agents are available and they are in very early stages of development [72]
TWIST1	Twist-related protein 1	1.51	3.56E−02	TWIST1 is significantly overexpressed in advanced stages of OSCC and its expression predicted LNM and poor patient survival [73, 74]	A naturally occurring alkaloid harmine has recently been shown to promote TWIST1 degradation [75]

## Discussion

Due to the high morbidity associated with the current clinical management of OSCC [1, 3], it is essential to identify putative oncogenes driving carcinogenesis that may also be used as prognostic factors and targeted for therapeutic benefit. Recently, the advancement of high-throughput multi-omic technologies has facilitated comprehensive molecular profiling of tumour samples to identify drivers of oncogenesis and progression, which may lead to the development of precision oncotherapeutics [32, 33]. Here, we analysed OSCC genomes and transcriptomes to identify candidate driver oncogenes on the chromosomal cytobands 3q22-3q29, which is frequently amplified in squamous cell carcinomas [14–17]. Using an intuitive filtering technique to analyse TCGA and other publicly available datasets, we identified two genes located on 3q22-3q29—*NCBP2, TFRC*—with potential clinical relevance in HPV-negative OSCC. Leveraging data from multiple datasets of OSCC patients increases the validity of our findings (Fig. 1A). Both *NCBP2* and *TFRC* were found to be amplified and overexpressed in OSCC compared to normal oral cavity squamous epithelium, with increased expression of both genes associated with worse prognosis (Figs. 1 and 2). Given that TCGA lacks protein expression data for these biomarkers, we sought to assess the clinical relevance of NCBP2 and TFRC proteins using IHC on TMAs associated with prospectively collected clinical data from an in-house cohort of OSCC patients. OSCC patient outcomes significantly differed by NCBP2 and TFRC levels, with higher protein expression scores associated with worse OS, DSS and PFI (Fig. 4). Multivariate Cox regression analysis suggests that NCBP2 and TFRC are independent prognostic factors in OSCC and can provide prognostic value in addition to the currently used TNM staging system (Table 3). Any differences in the survival analyses between TCGA and our prospective TMA cohort likely reflect the lack of correlation between mRNA and protein expression. We have also demonstrated that the *NCBP2* and *TFRC* expression is correlated at the mRNA level, but not at the protein level. This difference could be explained in part by the loss of sensitivity in evaluating protein expression semi-quantitatively via IHC, which may mask the underlying correlation between NCBP2 and TFRC levels. Furthermore, NCBP2 and TFRC may be regulated post-transcriptionally or post-translationally in different ways, reducing the correlation between these proteins compared to the correlation in gene expression.

Collectively, our results provide substantial evidence for the role of *NCBP2* and *TFRC* as driver oncogenes in OSCC. Several cancer genomic and transcriptomic studies have associated increased *TFRC* expression with the prognosis of various cancers [30, 31, 34–36], including OSCC. However, there is very little known about the involvement of NCBP2 in carcinogenesis and progression [37]. Also, our study is the first to identify that the expression of *NCBP2* and *TFRC* genes is driven by amplification. Interestingly, the amplification of the entire 3q22-29 locus itself was not associated with prognosis (Fig. S4) while the expression of individual genes within this locus was associated with patient outcomes. This indicates that genes present on amplified chromosomal regions might be involved in complex cellular processes, the impact of which cannot be adequately captured by querying the amplification status of the region containing these genes.

Given the growing number of studies describing the prognostic value of TFRC in squamous cell carcinomas [30, 31, 34–36], we focused our attention on NCBP2 for further assessing effects on cancer aggressiveness. Our in vitro results also suggest that NCBP2 drives OSCC proliferation, migration, and invasion of OSCC cell lines, highlighting the potential for exploiting NCBP2 as a therapeutic target (Fig. 5).

It is also noteworthy that high TFRC expression was associated with higher pack-years smoked (Table 2). Other studies have noted that higher cigarette consumption is associated with poor prognosis and immunosuppression [38, 39]. Interestingly, our single-cell RNAseq analysis revealed significant expression of *TFRC* in dendritic cells and macrophages, whereas *NCBP2* expression was almost entirely restricted to tumour cells. Also, TFRC expression was observed in bone marrow samples from GTEx (Fig. 3B, D). Therefore, it may be useful to further investigate if TFRC regulates immune responses in the tumour microenvironment.

Our reported association between DAB IHC-based TFRC and NCBP2 protein expression score and poor survival further demonstrates that both TFRC and NCBP2 protein expression may be used as a prognostic marker in the clinical management of OSCC. DAB-based IHC staining is a cost-effective and commonly used tool in pathology. Therefore, our DAB IHC-based assay offers a clinically feasible way to measure biomarker expression in OSCC patients that is less complex than assessing multigene prognostic signatures [40]. These novel biomarkers may provide an additional prognostic tool to clinicians besides currently used tumour staging approaches, allowing for more informed treatment decisions. Other recent studies have also identified novel prognostic markers in oral cancers using IHC [7, 41, 42]. Thus, we propose TFRC and NCBP2 as novel additions to a growing body of potential prognostic biomarkers in OSCC.

We found that *NCBP2* expression was significantly upregulated in tumour cells compared to normal human tissue samples from the GTEx consortium, and other cells in the tumour microenvironment [29] (Fig. 3A, C). This provides a good therapeutic window to develop targeted therapies against NCBP2 that might be less toxic to normal cells. Nagai et al. previously demonstrated that a TFRC antibody inhibited the growth of OSCC tumours in a murine xenograft model [18]. However, since we detected TFRC expression in the bone marrow and immune cells in the tumour microenvironment (Fig. 3B, D), TFRC-targeting might be less desirable compared to ablating NCBP2. IHC-based assessment of NCBP2 protein levels could be employed as a companion diagnostic for potential NCBP2-targeting therapies, helping tailor treatment to patients whose tumours are driven by NCBP2. We also identified potential downstream effectors of NCBP2 in OSCC (Fig. 6), some of which may be targeted by available small molecule inhibitors (Table 4). Deploying such targeted therapeutics in the clinic could accelerate the progress in improving OSCC survival outcomes and help ameliorate the morbidity associated with current OSCC treatment regimens.

## Supplementary information

Supplementary Tables and Figure legends

Supplementary methods

Figure S1

Figure S2

Figure S3

Figure S4

Figure S5

## Data Availability

Materials described in the manuscript, including all relevant raw data, will be freely available to any researcher wishing to use them for non-commercial purposes, without breaching participant confidentiality.
