# Supplementary material for: NCBP2 and TFRC are novel prognostic biomarkers in oral squamous cell carcinoma
Source: Cancer Gene Ther. 2023 Jan 12;30(5):752–65. doi: 10.1038/s41417-022-00578-8 (PMC10191846; doi:10.1038/s41417-022-00578-8)
Supplement: Supplementary file 1 — Supplementary Tables and Figure legends [file 41417_2022_578_MOESM1_ESM.docx]

**Supplementary Figures and Tables**

Figure S1: Mouse liver slides and human tissue microarrays following IHC staining with different concentrations of primary antibody targeting NCBP2 (A) and TFRC (B). All slides displayed at 40x magnification and scale bars indicate 50μm.

Figure S2: Boxplots showing the expression of each of *GMPS, RFC4, TFRC, and NCBP2* to be significantly upregulated in 3q22-3q29 patient samples as compared to non-amplified samples in the TCGA OSCC HPV-negative cohort.

Figure S3: 3q22-3q29 Amplification status is not significantly associated with OS (A), DSS (B) or PFI (C) in TCGA HPV-negative OSCC patients.

Figure S4: Scatter plots showing the expression of *NCBP2, TFRC, RFC4,* and *GMPS* to be not correlated to the mean promoter methylation beta value in TCGA HPV-negative OSCC patients.

Figure S5: Scatter plot showing the expression of *TFRC* and *NCBP2* to be positively correlated to each other (*Spearman ρ* (R) = 0.68, p < 2.2e-16) in TCGA HPV-negative OSCC patients.

Table S1: Studies chosen for differential expression analysis

| **Dataset/Study** | **Number of Tumour samples** | **Number of normal samples** |
| --- | --- | --- |
| GSE3524 (Toruner *et. al.* 2004) | 16 | 4 |
| GSE9844 (Ye *et. al.* 2008) | 26 | 38 |
| GSE30784 (Chen *et. al.* 2008) | 167 | 45 |
| GSE13601 (Estilo *et. al.* 2009) | 31 | 27 |
| GSE31056 (Reis *et. al.* 2011) | 23 | 25 |
| TCGA HPV-negative OSCC | 275 | 26 |

Table S2: Spearman Correlation (ρ) of individual CpG methylations in the promoters of respective genes and their mRNA expression (log_2_TPM).

| NCBP2 | | | | |
| --- | --- | --- | --- | --- |
| Promoter: Chromosome 3: 196,940,400-196,944,801 | | | | |
| Probe | Start | End | ρ | p-value |
| cg03306972 | 196941627 | 196941628 | -0.089 | 0.16503 |
| cg03689283 | 196941785 | 196941786 | 0.03 | 0.62292 |
| cg08493356 | 196941884 | 196941885 | 0.015 | 0.81039 |
| cg02672678 | 196942263 | 196942264 | 0.003 | 0.95607 |
| cg08424876 | 196942324 | 196942325 | 0.038 | 0.53675 |
| cg17036441 | 196942346 | 196942347 | 0.041 | 0.50746 |
| cg14187685 | 196942401 | 196942402 | -0.018 | 0.76604 |
| cg06219926 | 196942531 | 196942532 | 0.086 | 0.15742 |
| cg18746364 | 196942551 | 196942552 | 0.044 | 0.47277 |
| cg06172138 | 196942585 | 196942586 | 0.026 | 0.67429 |
| TFRC | | | | |
| Promoter: Chromosome 3: 196,078,400-196,083,401 | | | | |
| Probe | Start | End | Rho | p-value |
| cg17380870 | 196081731 | 196081732 | 0.015 | 0.81139 |
| cg14641705 | 196082008 | 196082009 | 0.054 | 0.38119 |
| cg25276849 | 196082048 | 196082049 | 0.062 | 0.31464 |
| cg27335386 | 196082050 | 196082051 | -0.031 | 0.60921 |
| cg21494636 | 196082115 | 196082116 | 0.056 | 0.36267 |
| cg26126750 | 196082327 | 196082328 | -0.003 | 0.96567 |
| cg24870846 | 196082380 | 196082381 | 0.038 | 0.53088 |
| cg22956956 | 196082484 | 196082485 | -0.012 | 0.8391 |
| cg11087101 | 196082722 | 196082723 | 0.098 | 0.65602 |
| RFC4 | | | | |
| Promoter: Chromosome 3: 186,805,000-186,807,601 | | | | |
| Probe | Start | End | Rho | p-value |
| cg19251352 | 186806511 | 186806512 | 0.119 | 0.05135 |
| cg27099625 | 186806517 | 186806518 | -0.015 | 0.8035 |
| cg12820191 | 186806522 | 186806523 | 0.094 | 0.12288 |
| cg00799353 | 186806576 | 186806577 | 0.04 | 0.50887 |
| cg03434872 | 186806606 | 186806607 | -0.042 | 0.48816 |
| cg10767703 | 186806770 | 186806771 | 0.021 | 0.72587 |
| cg03446973 | 186806783 | 186806784 | 0.033 | 0.59117 |
| cg26099943 | 186806785 | 186806786 | -9E-04 | 0.98864 |
| cg27559724 | 186806890 | 186806891 | -0.053 | 0.39016 |
| cg25585523 | 186806904 | 186806905 | -0.057 | 0.35102 |
| cg08440162 | 186806961 | 186806962 | -0.024 | 0.68972 |
| cg12583908 | 186806988 | 186806989 | 0.117 | 0.05442 |
| cg03852656 | 186807435 | 186807436 | 0.065 | 0.28861 |
| GMPS | | | | |
| Promoter: Chromosome 3: 155,869,400-155,873,401 | | | | |
| Probe | Start | End | Rho | p-value |
| cg12075144 | 155870271 | 155870272 | 0.065 | 0.28618 |
| cg14616479 | 155870335 | 155870336 | 0.083 | 0.1751 |
| cg19761337 | 155870347 | 155870348 | 0.045 | 0.46136 |
| cg00801868 | 155870581 | 155870582 | 0.033 | 0.59548 |
| cg00696023 | 155870593 | 155870594 | 0.001 | 0.98621 |
| cg06064855 | 155870923 | 155870924 | 0.009 | 0.8773 |
| cg01843367 | 155871286 | 155871287 | -0.026 | 0.66853 |
| cg11496093 | 155871784 | 155871785 | 0.025 | 0.67905 |
| cg10689438 | 155872114 | 155872115 | -0.151 | 0.01329 |

Table S3: Crosstabulation of NCBP2 and TFRC protein expression levels among 188 OSCC patients in the ORI cohort as determined by IHC staining of TMA slides. Spearman ρ = 0.082, p = 0.283.

|  | | TFRC expression score | | | | Total |
| --- | --- | --- | --- | --- | --- | --- |
|  |  | 0 | 1 | 2 | 3 |  |
| NCBP2 expression score | 0 | 2 | 0 | 0 | 1 | 3 |
|  | 1 | 3 | 19 | 17 | 7 | 46 |
|  | 2 | 4 | 27 | 32 | 11 | 74 |
|  | 3 | 2 | 19 | 21 | 10 | 52 |
| Total | | 11 | 65 | 70 | 29 | 175 |
