# Supplementary material for: NCBP2 and TFRC are novel prognostic biomarkers in oral squamous cell carcinoma
Source: Cancer Gene Ther. 2023 Jan 12;30(5):752–65. doi: 10.1038/s41417-022-00578-8 (PMC10191846; doi:10.1038/s41417-022-00578-8)
Supplement: Supplementary file 2 — Supplementary methods [file 41417_2022_578_MOESM2_ESM.docx]

Supplementary methods – TCGA OSCC Promoter DNA Methylation and mRNA Gene Expression Analysis

R studio (version 4.2.0) and R package TCGAbiolinks (version 2.24.3) were used to analyze a cohort of 275 TCGA OSCC samples. TCGAbiolinks was used to download DNA methylation beta values (Illumina Human Methylation 450 array) and transcriptome profiling mRNAseq counts for all 275 TCGA OSCC samples. DNA methylation beta values and mRNAseq counts were filtered for 4 genes (NCBP2, TFRC, RFC4, and GMPS). mRNAseq raw counts were normalized to TPM values and converted to log2 scale. CpG promoter probes for all four genes were identified and filtered for by genomic base pair position. R function cor.test was used to calculate spearman correlation values between CpG promoter probes and log2TPM(mRNA) counts. Firstly, individual CpG promoter probes were correlated with log2TPM(mRNA) counts, rho and p-values are reported. Secondly, CpG promoter beta values were averaged across all TCGA OSCC samples and correlated with log2TPM(mRNA) counts. Scatter plots of mean promoter beta value verses log2TPM(mRNA) counts are shown for the four genes analyzed.
