# Supplementary figures and images for: NCBP2 and TFRC are novel prognostic biomarkers in oral squamous cell carcinoma
Source: Cancer Gene Ther. 2023 Jan 12;30(5):752–65. doi: 10.1038/s41417-022-00578-8 (PMC10191846; doi:10.1038/s41417-022-00578-8)

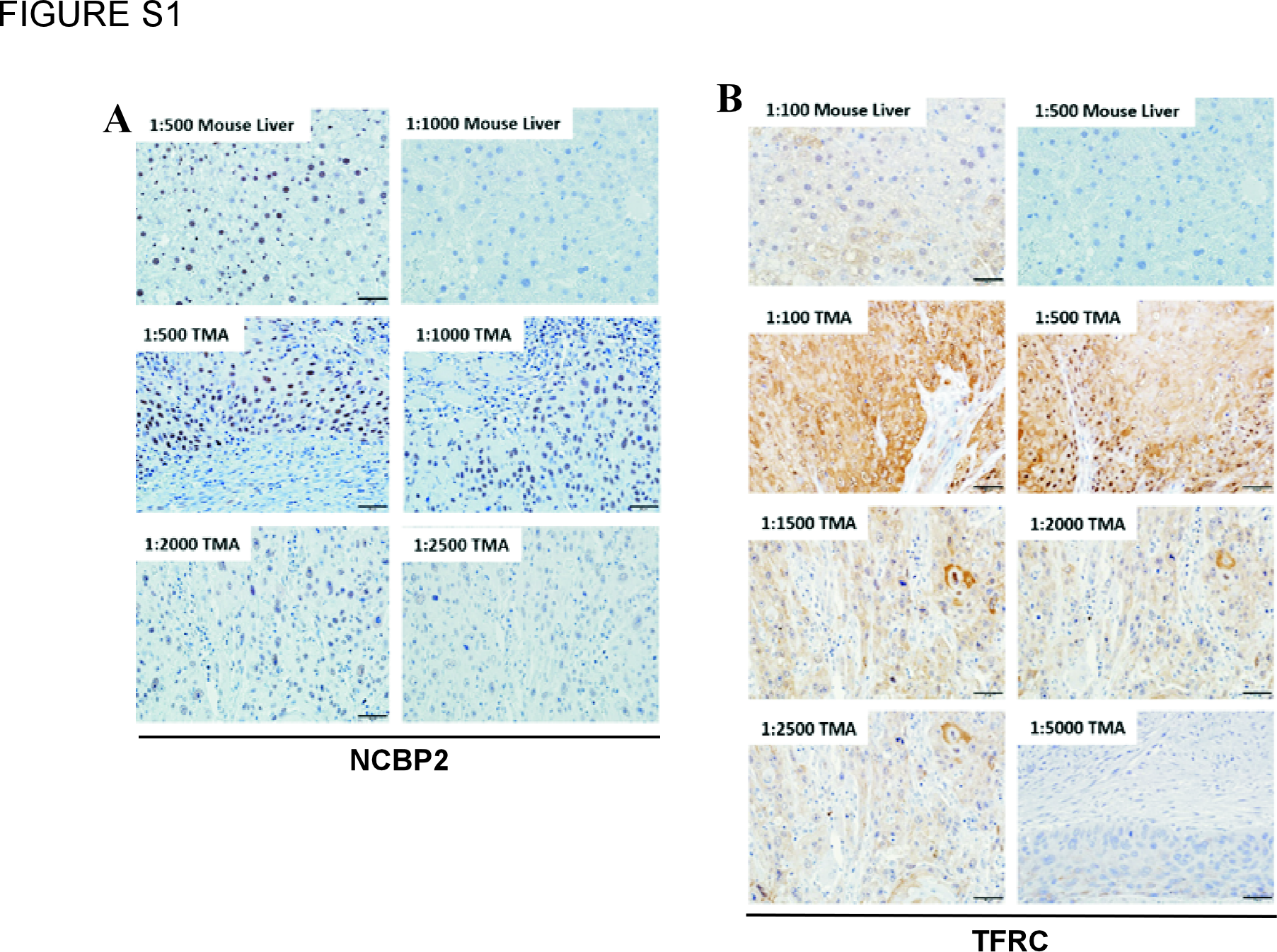

Supplement: Supplementary file 3 — Figure S1 [file 41417_2022_578_MOESM3_ESM.tif]

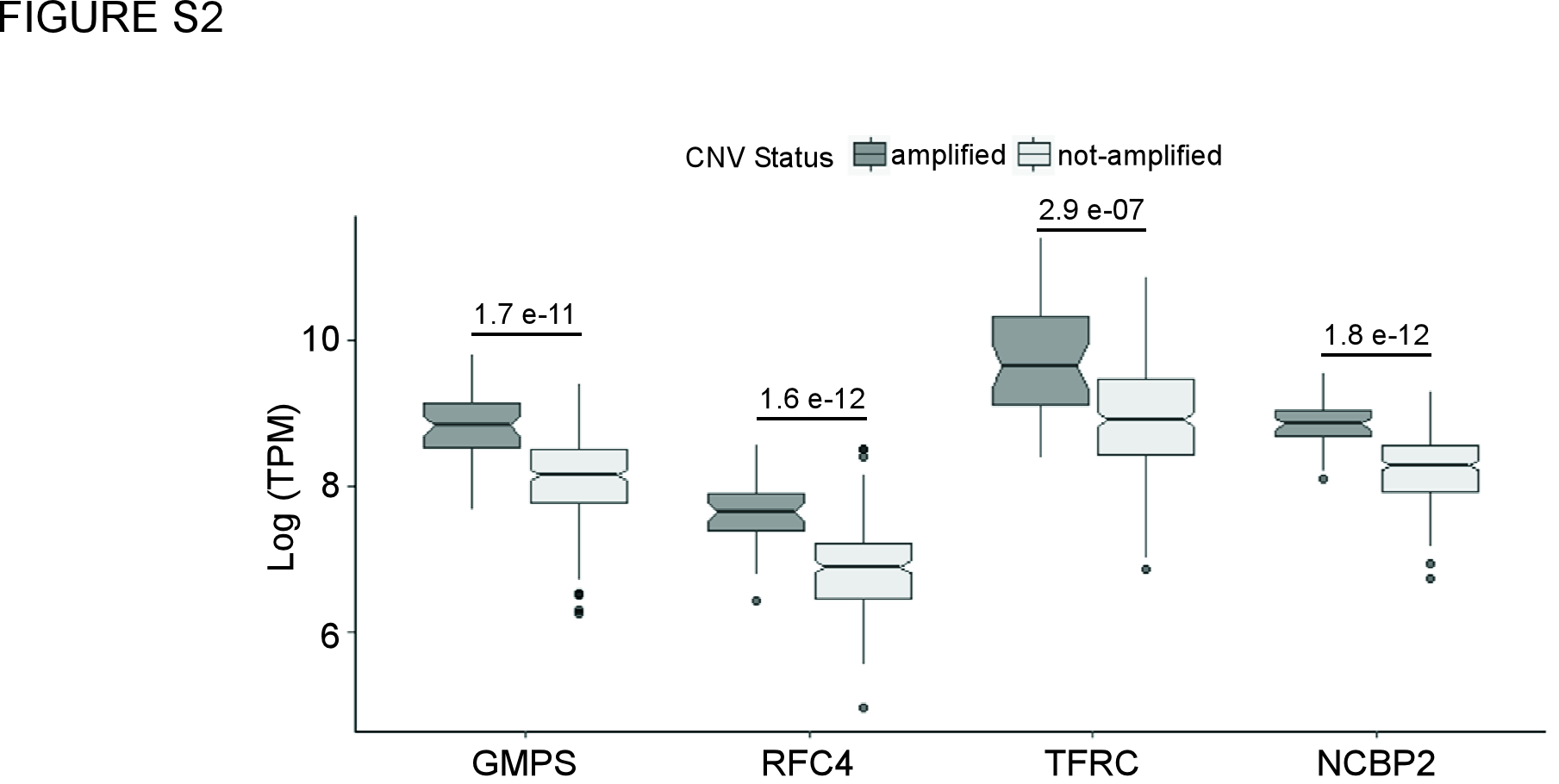

Supplement: Supplementary file 4 — Figure S2 [file 41417_2022_578_MOESM4_ESM.tif]

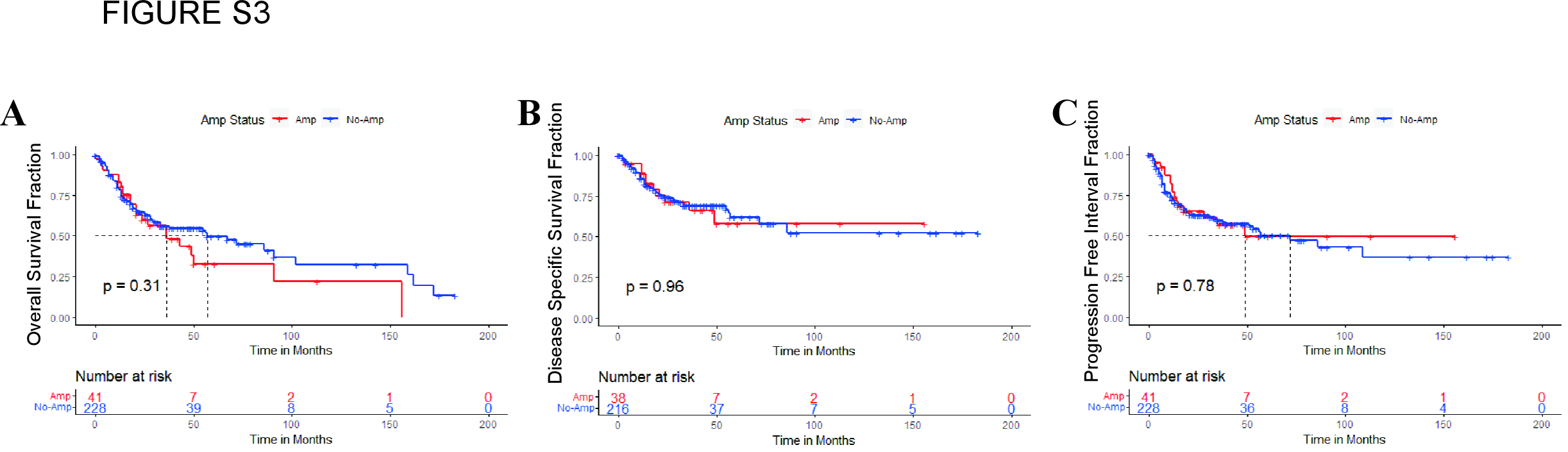

Supplement: Supplementary file 5 — Figure S3 [file 41417_2022_578_MOESM5_ESM.tif]

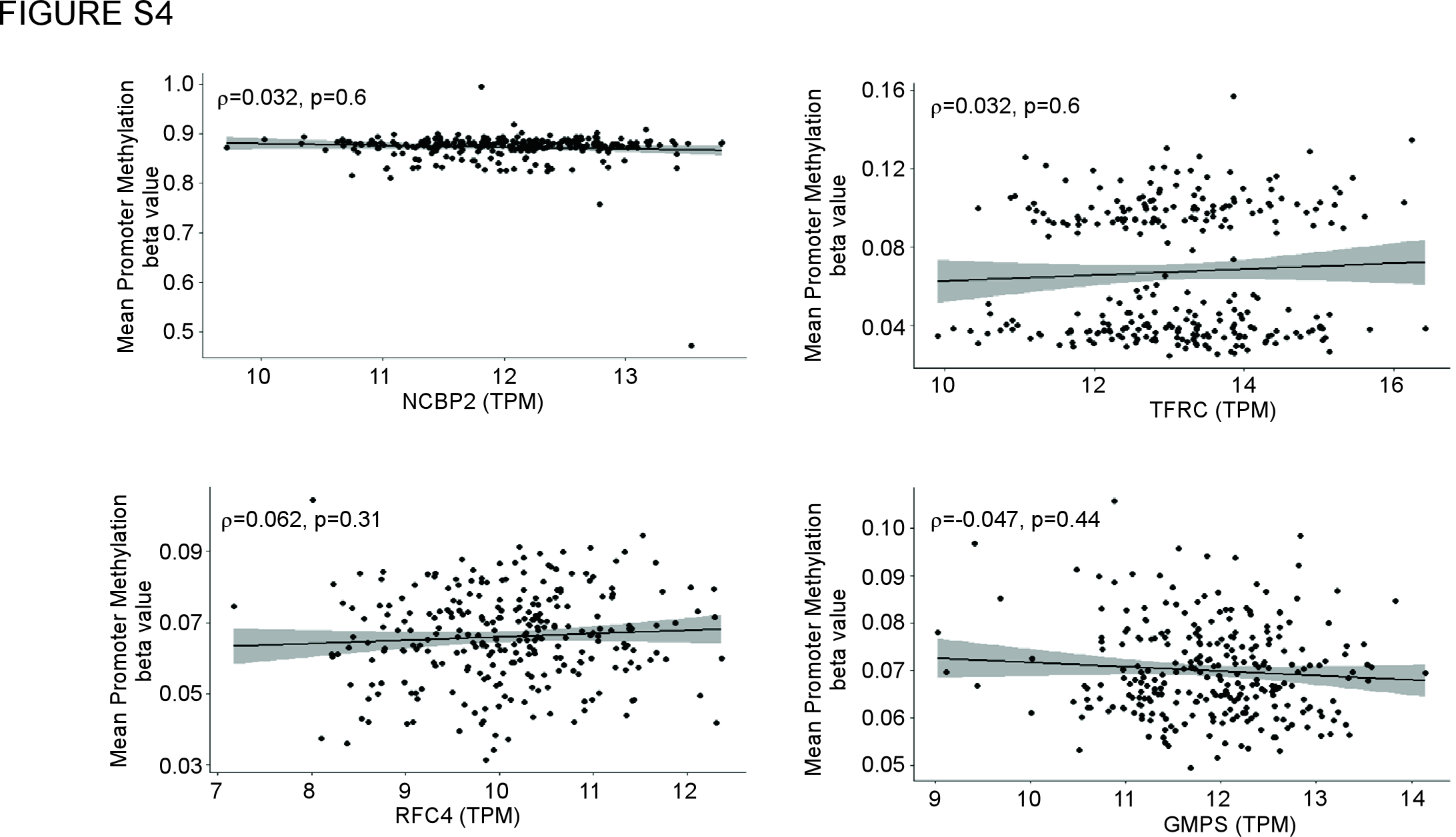

Supplement: Supplementary file 6 — Figure S4 [file 41417_2022_578_MOESM6_ESM.tif]

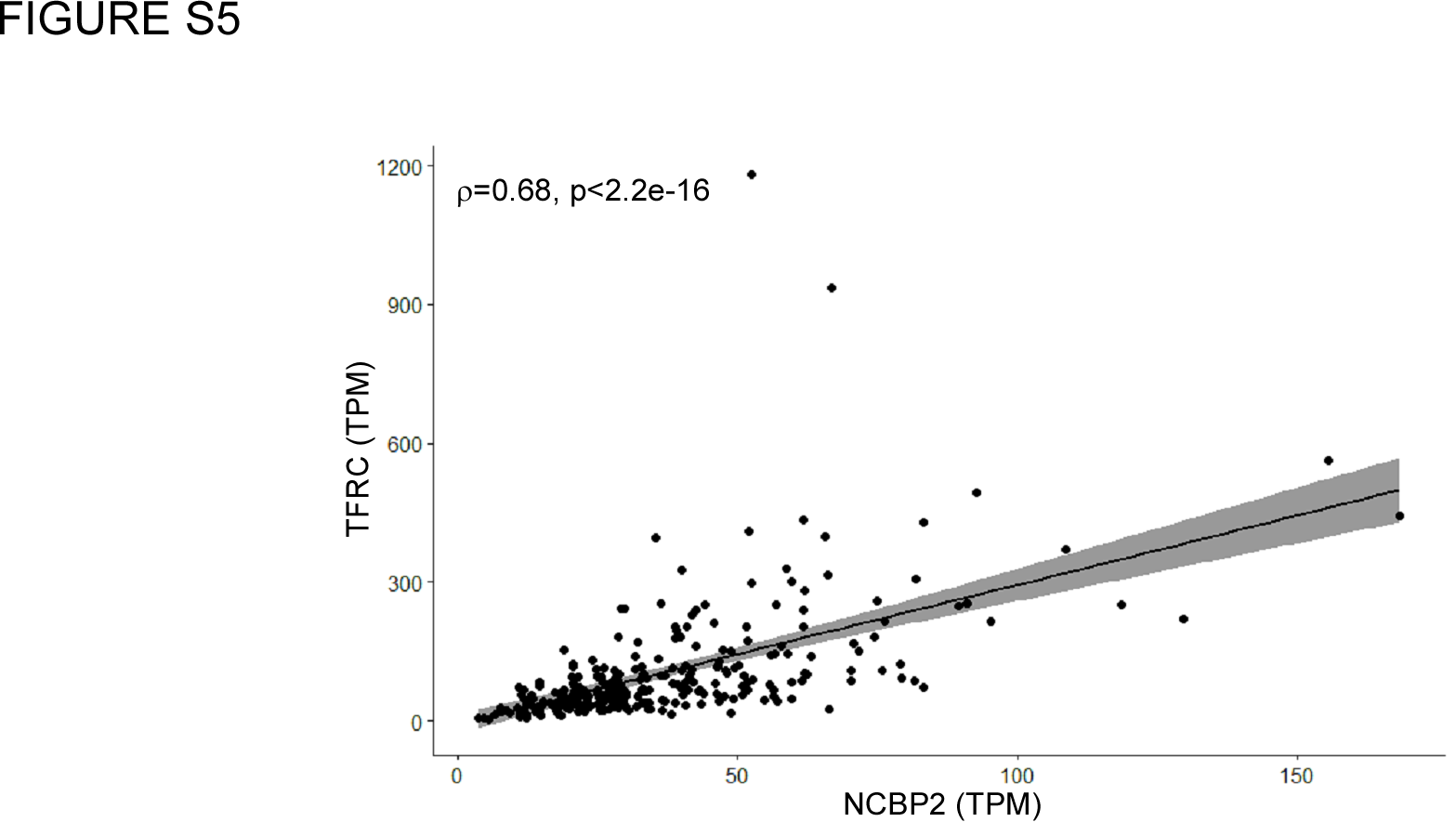

Supplement: Supplementary file 7 — Figure S5 [file 41417_2022_578_MOESM7_ESM.tif]
